# Analysis of Metal Contents in Portland Type V and MTA-Based Cements

**DOI:** 10.1155/2014/983728

**Published:** 2014-11-09

**Authors:** Maura Cristiane Gonçales Orçati Dorileo, Matheus Coelho Bandeca, Fábio Luis Miranda Pedro, Luiz Evaristo Ricci Volpato, Orlando Aguirre Guedes, Ricardo Dalla Villa, Mateus Rodrigues Tonetto, Alvaro Henrique Borges

**Affiliations:** ^1^Faculty of Dentistry, University of Cuiaba, Avenida Manoel Jose de Arruda 3.100, Jardim Europa, 78065-900 Cuiaba, MT, Brazil; ^2^Master's Program in Dentistry, UNICEUMA, Rua Josue Montello 01, Renascenca, 65075-120 São Luıs, MA, Brazil; ^3^Master's Program in Chemistry, Mato Grosso Federal University, Avenida Fernando Correa da Costa 2367, Boa Esperanca, 78060-900 Cuiaba, MT, Brazil; ^4^Master's Program in Integrated Dentistry Science, Faculty of Dentistry, University of Cuiaba, Avenida Manoel Jose de Arruda 3.100, Jardim Europa, 78065-900 Cuiaba, MT, Brazil

## Abstract

The aim of this study was to determine, by Atomic Absorption Spectrometry (AAS), the concentration levels of 11 metals in Type V gray and structural white PC, ProRoot MTA, and MTA Bio. Samples, containing one gram of each tested cement, were prepared and transferred to a 100 mL Teflon tube with a mixture of 7.0 mL of nitric acid and 21 mL of hydrochloric acid. After the reaction, the mixture was filtered and then volumed to 50 mL of distilled water. For each metal, specific patterns were determined from universal standards. Arsenic quantification was performed by hydride generator. The analysis was performed five times and the data were statistically analyzed at 5% level of significance. Only the cadmium presented concentration levels of values lower than the quantification limit of the device. The AAS analysis showed increased levels of calcium, nickel, and zinc in structural white PC. Type V PC presented the greatest concentration levels of arsenic, chromium, copper, iron, lead, and manganese (*P* < 0.05). Bismuth was found in all cements, and the lowest concentration levels were observed in Portland cements, while the highest were observed in ProRoot MTA. Both PC and MTA-based cements showed evidence of metals inclusion.

## 1. Introduction

During the different phases of the endodontic treatment, accidents and complications can occur as a consequence of the complex inner dental anatomy, lack of knowledge of the mechanical properties of instruments, inadequate technical sequences, and lack of professional ability [1]. The most frequent complications are related to root canal deviations (apical transportation), instrument breakage, and radicular perforation [1, 2]. For treatment in cases of perforation, the access can occur by endodontic procedures, in an orthograde manner, or by surgical procedures, in the external tooth root area [1–4]. Apicectomy with retrograde filling is defined as apical root resection, followed by cavity confection and subsequent filling with a proper material [5]. In 1993, at the University of Loma Linda in the United States, a new MTA- (Mineral Trioxide Aggregate-) based cement, indicated for sealing perforating areas in human teeth, was developed. The results showed excellent physical [6–8], chemical [9, 10], and biological properties [3, 4].

MTA was initially described as a primary derivative of calcium oxide, silicate dioxide, and aluminum oxide, which during the production process resulted in combinations of tricalcium silicate, dicalcium silicate, and tricalcium aluminate [6, 7, 10]. In 1999, MTA was evaluated and approved by the Food and Drug Administration, and since then, it has been commercially available as ProRoot MTA (Tulsa Dental Products, Tulsa, OK, USA) [9]. Subsequently, it became clear that this material was Portland cement (PC) with greater finesse and the presence of bismuth oxide as radiopacifying agent [11]. In 2001, Dentsply Tulsa revealed that the MTA composition of PC was 75%, as well as 20% bismuth oxide and 5% dihydrate calcium sulfate. At the same time, MTA Angelus, described as 80% PC and 20% bismuth oxide [12], was introduced on the Brazilian market. Currently, MTA cement is found commercially in two formulations and is available in gray and white versions [9].

Based on the biocompatibility [3, 4] and similar compositions of MTA-based cements [11], pure PC has been proposed to be potential substitute to MTA in endodontic procedures [5–8]. It is known that the main compound of MTA-based cements is the ordinary Type I PC [5, 8]. However, Type I PC has low resistance to compression [13], and the addition of bismuth oxide to MTA increases its porosity and friability over time [14]. Based on different types of PC, other materials should be studied in order to certify the alternatives as matrix for the MTA-based cements. Type V PC receives a special treatment during the production, resulting in a material with higher resistance compared to other cements [15]. This material has different specifications of limestone and clay. It receives a special grilling treatment during the production of the clinker. As a result, the finest particles are formed, offering high resistance for the material in a shorter period of time, compared to other cements [15].

The PC has high resistance to compression in consequence of the presence of additives in its composition [13]. The biocompatibility of Type V PC had been proved in animal studies by the observation of biomineralization areas [3, 4]. On the other hand, studies have shown the presence of contaminant metals, which were aggregated during the production process as a result of the substitution of the primary fuel by alternative fuels [16–18]. Heavy metals originate from the use of waste materials as inorganic raw material or from secondary fuels used to fire the kiln during cement manufacture [18]. In humans, the presence of contaminant elements can alter important organic functions by intoxication and increases the risk of developing diseases such as cancer, alteration of neurological functions, hepatic disorders, renal disorders, and auditory loss, as well as, in some cases, death by intoxication [19]. Thus, the present investigation aimed to quantify, by atomic absorption spectrophotometry, the concentrations of metals in structural white PC, Type V gray PC, ProRoot MTA, and MTA Bio.

## 2. Material and Methods

The materials evaluated in the present study and their compositions are described in Table 1. All materials were manipulated in accordance with the manufacturer's instructions.

Total metal content was determined according to the ISO 9917-1 [20] method. The metals analyzed were arsenic, bismuth, cadmium, calcium, chromium, copper, iron, lead, manganese, nickel, and zinc.

Five samples were prepared for each tested cement. One gram of each sample, to the nearest 0.001 g, was weighed and transferred to a 100 mL Teflon tube. A mixture of 7.0 mL nitric acid (65% by volume) and 21 mL hydrochloric acid (37% by volume) was added to the Teflon tube and allowed to stand for 2 hours. After this period of time, the vessel was loosely capped and transferred to a hood containing an exhaust vent, where it was heated to 80°C by placing it on a heating block and was then allowed to equilibrate for 150 mins. After the reaction vessel was cooled to room temperature, the mixture was filtered with paper filter number 40 (Whatman, GE, Healthcare Life Sciences, Pittsburgh, PA, USA) to obtain a filtrate and then the volume was completed to 50 mL of distilled water. A blank test was performed in parallel by the same procedure with the use of the same quantities of all reagents but omitting the test sample. For each metal, specific patterns were determined from universal standards (Merck, Darmstadt, Germany), as it is described in Table 2. Micropipettes (Boeco, Germany) with adjustable volumes of 5–50 *μ*L, 50–200 *μ*L, and 100–1000 *μ*L were used for the preparation of the standard solutions and samples.

The standards were prepared by addition of appropriate amounts of aqueous analyte stock solutions plus 1.000 g of washed cement, to 10.00 mL volumetric flasks, which were completed with bidistilled water. The bidistilled water was used for adjustment and the washing procedure was performed in a 250 mL separating funnel to which were added 50.0 mL of bidistilled water and 100.0 mL of a solution of 1.0% (v/v) nitric acid, heated at 70°C. The separating funnel was shaken vigorously for 5 mins, and the extraction procedure was repeated until no analytes were detected in the sample by flame atomic absorption spectrometer. Analytical curves were prepared in the concentration range 0.0–0.8 mg L^−1^, using the bidistilled water medium. Sample preparation essentially consisted of the addition of 1.000 g of bidistilled water to 10.00 mL volumetric flasks, which were completed with bidistilled water. All standards and samples were prepared immediately prior to analysis.

A Spectra AA220 flame atomic absorption spectrometer (Varian Ind. Com. Ltda., São Paulo, SP, Brazil) with aspiration tax of 2 mL/min and specific hollow cathode lamps was used for each metal determination. The lecture parameters of each analyzed element were determined according to the electric current, wavelength, and slit aperture of the device, as described in Table 3.

The arsenic quantification was performed by using an atomic absorption spectrophotometer Spectra A (Varian Ind. Com. Ltda., São Paulo, SP, Brazil) equipped with a hydride generator and 3 psi of hydrogen pressure, nitrogen in 50 mL/min, hydrochloric acid solution in 10 mol with aspiration tax of 1 mL/min, and 1% sodium borohydride in 1% sodium hydroxide (Merck, Darmstadt, Germany) with aspiration tax of 1 mL/min. The aspiration tax of the sample was 6 mL/min in 40 secs of integration time. The analyzed conditions were the following: arsenic hollow cathode lamp (Varian Ind. Com. Ltda., São Paulo, SP, Brazil) and operating at 193.7 nM wavelength with 10 mA lamp operation current and slit aperture of 0.5 nM. Arsenic patterns were made in concentrations of 0.1; 0.25; 0.5; 1; and 2 mg/L. All measurements were performed in five replications.

Statistical analyses were carried out using ANOVA and Tukey's test at 5% level of significance. When sample distribution was nonnormal, nonparametric analysis of variance was performed with Kruskal-Wallis test (*α* = .05). The tests were performed using IBM SPSS Statistical software, version 21.0 for Windows (IBM Corporation, Somers, NY, USA).

## 3. Results

Table 4 shows the means metal contents value, the standard deviations, and the differences between structural white PC, Type V gray PC, MTA Bio, and ProRoot MTA by using atomic absorption spectrophotometry.

All metals were detected in the tested cements. Cadmium, for both PC and MTA-based cements, presented values lower than the quantification limit of the device. The arsenic concentration was the highest in Type V gray PC (*P* < 0.05). MTA Bio and ProRoot MTA had similar concentration of arsenic, calcium, copper, lead, and zinc (*P* > 0.05). The concentration of arsenic, lead, copper, chromium, iron, and manganese in Type V gray PC was significantly higher than that in other materials (*P* < 0.05). Structural white PC had the highest nickel concentration. Bismuth was found in all cements, the lowest concentration levels were observed in Portland cements, while the highest in ProRoot MTA (*P* < 0.05).

## 4. Discussion

As a rule, the components of MTA-based cements should be described in the data sheet [9, 10, 16, 17]. The PC is commonly used in engineering and the raw materials for its basis are dependent from the material's origin [13, 15]. Usually, Type I PC is the main material of MTA-based cements [8–10, 14], but when new material is proposed for this end, the investigations should be conducted again. Type V PC can be used as alternative material to substitute MTA-based cements for clinical practice [3, 4]. However, more than understanding the behavior of these materials in the organism, for safe clinical use, it is necessary to study their compositions.

The chemical characterization of dental materials that are used in close contact with periapical tissues is a predictive factor to understand their physical, chemical, and biological properties [21, 22]. The knowledge of their chemical composition is crucial in choosing the best material to be used in clinical practice. Thus, the present study intended to determine the levels of metal contents of Portland Type V and MTA-based cements employing atomic spectrometry analysis.

Analyzing the materials, the main difference is related to the size and regularity of the particles and the exclusive presence of bismuth in MTA-based cements [16]. In MTA cements, the particles are more homogeneous and smaller, whereas in Portland cements, there are different sizes and widths [9, 10, 23, 24]. Regarding MTA Bio and ProRoot MTA, according to the distributions and particles sizes, these cements are similar to each other. Calcium oxide is one of the most important compounds in these cements [21]. Based on this fact, in this study, calcium ions were most commonly found, in accordance with the literature [9, 19, 21]. All the analyzed materials presented large amounts of calcium [21, 25], and the presence of this element is associated with calcium hydroxide formation during the hydration reaction [11, 23–25]. Calcium hydroxide is responsible for the alkalization and bioactive effects of these materials in vital tissues, contributing to the healing process [21].

Iron is primarily obtained from the minerals hematite and magnetite. The minerals taconite, limonite, and siderite are other important sources. Iron is an important chemical element present in nature in the form of Fe^+3^ ions, which are less soluble in water, and is essential for cellular homeostasis and chiefly for hemoglobin oxygen transportation in blood vessels [19]. Deficiency of iron leads to anemia, but excess iron in the body can cause liver and kidney damage. In the PC, iron improves the hardness of the final mass [15]. The iron toxicity for human consumption is above 20 milligrams for every kilogram of mass [19]. Type V PC presented the highest concentration of this element, and this result has also been supported by other studies [17, 18]. In contrast, it is important to note that the white PC, ProRoot MTA, and MTA Bio also presented this chemical element, although in low concentrations, in accordance with the literature [22].

As important as calcium and iron is the presence of copper, zinc, and chromium. The most familiar forms of copper are pure copper, brass (copper-zinc alloy), and bronze (copper-tin alloy). It is found in nature mostly in impure mineral forms. Copper participates in iron fixation to hemoglobin, synthesis of neurotransmitters, cardiac and vascular integrity, bone resistance, and neutrophil maturation. The minimum acceptable daily oral intake for copper is 0.020 mg/kg of body weight for adults and approximately 0.050 mg/kg body weight for infants. Zinc contributes to immune system functioning, and it is essential to wound healing and ADN synthesis [19]. Metallic zinc is not considered toxic, but some zinc compounds, such as oxide/sulfide, are harmful, and excessive absorbance of them can interfere with the organic absorbance of iron and copper. The levels of zinc that may produce adverse effects are much higher than 11 mg/day for men and 8 mg/day for women [19]. Chromium is fundamental for human life, although, in excess and in the oxidative form, it can cause problems in the respiratory system, mucosa, and skin, as well as lung cancer. In the trivalent form, naturally found in human organism, it is very stable and not dangerous to the patient's health. In its quadrivalent form, found as a result of industrial processing during manufacturing and in vitamin supplements, it is potentially carcinogenic. Acute oral toxicity ranges between 1.5 and 3.3 mg/kg [19]. Type V PC presented the highest concentration of all these three elements, in agreement with previous studies [17, 18]. Regarding the presence of chromium, ProRoot MTA was considered safe for human use [16].

The heavy metals analyzed in this study were arsenic, bismuth, cadmium, lead, manganese, and nickel. Only cadmium was identified in concentrations lower than the instrument quantification limit (0.002 mg/kg), which is safe when the tolerable concentration is considered (0.2 mg/kg) [19]. Regarding lead, the concentration levels were, in all the cements, lower than the maximum dose recommended (1.5 mg/kg) [20]. Comparing the cements, no differences were among them, in accordance with Camilleri et al. (2012) [18]. Chang et al. (2010) [17] detected the presence of this metal only in PC. After iron, manganese is the second most abundant metal in nature, and it plays important role in life, considering its structural and enzymatic functions. In this study, all the cements presented concentrations lower than toxic levels (300 *μ*mol/kg) [19]. The highest concentration was observed in Type V PC; similar results were found in the literature [24]. Nickel forms free radicals in humans through the oxidation and reduction reactions (redox) of Ni^+3^ to Ni^+2^ [19]. The highest concentration was observed in structural white PC, although a high concentration was also observed in Type V PC as a result of the enrichment of this material with the chemical element during the manufacturing process, aiming at greater resistance to corrosion [15]. MTA-based cement presented lower concentrations, as it was observed previously [17].

Bismuth has a low toxicity potential as a result of its low absorbance by humans and it is found freely in nature, as well as in minerals such as bismuthine and in bismuth oxide [19]. In dentistry, this element is added to improve the radiopacity of the material to be distinguished from surrounding structures and to reveal empty spaces and inappropriate contours [14, 24], but it can alter physicochemical properties, such as setting time [8, 11], hydration reaction [10, 23, 24], and porosity and density [14]. In humans, the normal blood level is on average less than 1 ug/dL while poisoned subjects average 3 ug/dL [19]. All the cements presented concentrations levels of this element, even in low quantities, as was presented in Type V PC and structural white PC, in disagreement with many studies in literature, which have reported that bismuth was exclusively present in MTA-based cements [11, 26]. The greatest concentrations were observed in MTA-based cements, which were related to the production process during which the element is added to the material to improve material's radiopacity [24, 26].

Arsenic is a metalloid encountered in nature in both inorganic and organic forms and in different stages of oxidation. It can inhibit cellular respiration, alter genetic expression of stress proteins, and react with sulfhydryl groups to deactivate proteins and enzymes [19]. Trivalent and pentavalent stages of oxidation are the most toxic stages, and arsenic also shows carcinogenic effects, leading the inhibition of DNA repair and, as a result, mutations at key genetic sites or increases in cell proliferation [27]. The results of the present study showed very low levels of arsenic released by the materials. Type V PC presented the highest concentration of this element but in lower levels than those recommended as safe by ISO 9917-1 (2007) [20], that is, 2 mg/kg. No differences were observed between the MTA-based cements, and arsenic appeared in safe concentrations for human use. The greater amount of arsenic in PC and regular MTA could be attributed to differences in product manufacturing. These results are in accordance with the literature [12, 16].

The results of the present study lead to a better understanding of the interactions that occur between these MTA-based and Portland cements and periapical tissues. This extended comprehension should help investigators to design new products, with well-defined characteristics.

## 5. Conclusion

In the present study, all the metals (arsenic, bismuth, cadmium, calcium, chromium, copper, iron, lead, manganese, nickel, and zinc) were detected in PC and MTA-based cements. Only cadmium concentration levels were lower than the quantification limit of the device. All the analyzed materials presented large amounts of calcium with the greatest concentration level of this metal in structural white PC. Type V PC had significantly more arsenic, chromium, copper, iron, lead, and manganese. The highest concentration levels of nickel and zinc were present in structural white PC. Bismuth was present in higher concentration in MTA-based cements than Portland cements. Type V Portland cement may be considered a matrix for the development of new root-filling materials. However, further physicochemical and biological tests should be conducted.

## Figures and Tables

**Table 1:** Composition of the materials and their manufacturers.

Cement	Composition (MSDS data)	Manufactures
Structural white PC	White clinker (100–75%), gypsum (3%), and carbonate material (0–25%)	Votorantim Cimentos, São Paulo, SP, Brazil

Type V gray PC	Clinker and gypsum (100–95%) and carbonate material (0–5%)	Votorantim Cimentos, São Paulo, SP, Brazil

MTA Bio	Portland cement (80%) and bismuth oxide (20%)	Ângelus Ind. Prod., Londrina, PR, Brazil

ProRoot MTA	Portland cement (75%), bismuth oxide (20%), and gypsum (5%)	Dentsply-Tulsa Dental, Tulsa, OK, USA

**Table 2:** Universal standards concentration (mg/L) for analyzed metals.

Metals	Universal standard				
	1	2	3	4	5
Bismuth	0.10	0.50	1.00	2.00	4.00
Cadmium	0.10	0.50	1.00	2.00	3.00
Calcium	0.15	0.25	0.50	1.00	2.00
Chromium	0.10	0.50	1.00	2.00	4.00
Copper	0.10	0.50	1.00	2.00	4.00
Iron	0.10	0.50	1.00	2.00	4.00
Lead	0.10	0.50	1.00	2.00	4.00
Manganese	0.10	0.50	1.00	2.00	4.00
Nickel	0.10	0.50	1.00	1.50	2.00
Zinc	0.50	1.00	2.00	4.00	—

**Table 3:** Instrumental operating conditions.

Metals	Current (mA)	Wave length (nM)	Slit aperture (nM)
Bismuth	12.0	223.1	0.2
Cadmium	4.0	228.8	0.5
Calcium	10.0	422.7	0.5
Chromium	7.0	357.9	0.2
Copper	4.0	324.8	0.5
Iron	5.0	248.3	0.2
Lead	10.0	217.0	1.0
Manganese	5.0	279.5	0.2
Nickel	4.0	232.0	0.2
Zinc	5.0	213.9	1.0

**Table 4:** Means and standard deviations of metal contents (mg/kg) for the tested materials by using atomic absorption spectrophotometry.

Metal	Cement			
	Structural white PC	Type V gray PC	MTA Bio	ProRoot MTA
Arsenic	3.70 ± 0.10^b^	5.40 ± 0.10^a^	3.05 ± 0.10^c^	2.80 ± 0.20^c^
Bismuth	240.00 ± 10.00^d^	80.00 ± 10.00^c^	57.400.00 ± 7.230.00^b^	97.630.00 ± 30.970.00^a^
Cadmium	^*^ <QL	^*^ <QL	^*^ <QL	^*^ <QL
Calcium	316.380.00 ± 31.370.00^a^	240.220.00 ± 10.140^b^	248.710.00 ± 21.370.00^b^	214.870.00 ± 26.100.00^b^
Chromium	25.00 ± 4.00^c^	47.00 ± 1.00^a^	36.00 ± 2.00^b^	16.00 ± 1.00^d^
Copper	5.00 ± 0.00^b^	6.00 ± 0.00^a^	5.00 ± 0.00^b^	5.00 ± 0.00^b^
Iron	740.00 ± 40.00^c^	6.450.00 ± 540.00^a^	1.380.00 ± 90.00^b^	650.00 ± 80.00^c^
Lead	30.00 ± 00.00^b^	40.00 ± 0.00^a^	30.00 ± 00.00^b^	30.00 ± 0.00^b^
Manganese	55.00 ± 3.00^d^	314.00 ± 15.00^a^	66.00 ± 1.00^c^	82.00 ± 7.00^b^
Nickel	30.00 ± 0.00^a^	20.00 ± 0.00^b^	18.00 ± 0.00^c^	12.00 ± 0.00^d^
Zinc	130.00 ± 10.00^a^	120.00 ± 00.00^b^	30.00 ± 10.00^c^	10.00 ± 0.00^c^

^*^ <QL lower than the quantification limit of the device (0.002 mg/kg).

Superscript lower case letters compare groups in horizontal lines. Different letters represent statistically significant difference (*P* < 0.05).
